# These legs were made for propulsion: advancing the diagnosis and treatment of post-stroke propulsion deficits

**DOI:** 10.1186/s12984-020-00747-6

**Published:** 2020-10-21

**Authors:** Louis N. Awad, Michael D. Lewek, Trisha M. Kesar, Jason R. Franz, Mark G. Bowden

**Affiliations:** 1 College of Health and Rehabilitation Sciences: Sargent College, Boston University, Boston, MA USA; 2 Division of Physical Therapy, Department of Allied Health Sciences, University of North Carolina at Chapel Hill, Chapel Hill, NC USA; 3 Division of Physical Therapy, Emory University, Atlanta, GA USA; 4 Joint Department of Biomedical Engineering, University of North Carolina at Chapel Hill and North Carolina State University, Chapel Hill, NC USA; 5 Division of Physical Therapy, Medical University of South Carolina, Charleston, SC USA

**Keywords:** Propulsion, Locomotion, Walking, Rehabilitation, Diagnosis, Intervention, Sensors, Robotics

## Abstract

Advances in medical diagnosis and treatment have facilitated the emergence of precision medicine. In contrast, locomotor rehabilitation for individuals with acquired neuromotor injuries remains limited by the dearth of (i) diagnostic approaches that can identify the specific neuromuscular, biomechanical, and clinical deficits underlying impaired locomotion and (ii) evidence-based, targeted treatments. In particular, impaired propulsion by the paretic limb is a major contributor to walking-related disability after stroke; however, few interventions have been able to target deficits in propulsion effectively and in a manner that reduces walking disability. Indeed, the weakness and impaired control that is characteristic of post-stroke hemiparesis leads to heterogeneous deficits that impair paretic propulsion and contribute to a slow, metabolically-expensive, and unstable gait. Current rehabilitation paradigms emphasize the rapid attainment of walking independence, not the restoration of normal propulsion function. Although walking independence is an important goal for stroke survivors, independence achieved via compensatory strategies may prevent the recovery of propulsion needed for the fast, economical, and stable gait that is characteristic of healthy bipedal locomotion. We posit that post-stroke rehabilitation should aim to promote independent walking, in part, through the acquisition of enhanced propulsion. In this expert review, we present the biomechanical and functional consequences of post-stroke propulsion deficits, review advances in our understanding of the nature of post-stroke propulsion impairment, and discuss emerging diagnostic and treatment approaches that have the potential to facilitate new rehabilitation paradigms targeting propulsion restoration.

## Introduction

THE fast, economical, and stable gait that is characteristic of healthy bipedal locomotion [1–6] requires the coordination of three locomotor subtasks—propulsion, limb advancement, and bodyweight support. During the propulsion locomotor subtask, positive work by the trailing limb accelerates the body into the next gait cycle [7]. To walk faster, people with intact neural control symmetrically increase the positive work performed by each limb [8–10]. The coordinated modulation of the work performed by each limb leverages the natural oscillatory dynamics that arise from repeating foot-ground interactions to optimize stability and economy of effort while regulating walking speed [6, 10]. In contrast, the hemiparetic gait observed after stroke [11–13] is slow [14–17], metabolically expensive [10, 15, 18–20], and unstable [21–24]. In neurologically unimpaired individuals, the plantarflexor muscles are the primary generators of positive work [9]; however, post-stroke neuromotor deficits result in a distal-to-proximal redistribution of the positive work generated by the muscles of the paretic limb [10, 25, 26], and, ultimately, a markedly altered profile for the anterior ground reaction force (i.e., the propulsion force) [27].

**Fig. 1:**
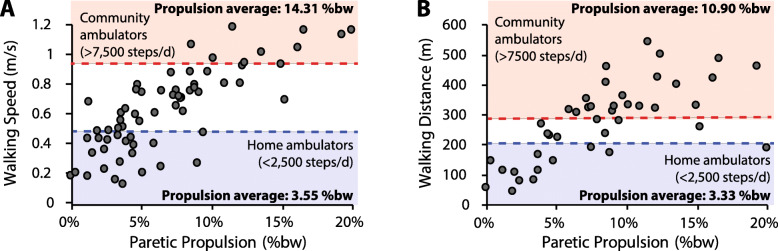
Relationship between peak paretic propulsion and walking **a** speed and **b** distance. Speeds and distances indicative of unlimited community ambulation are in red. Those indicative of home ambulation are in blue. See [17, 28, 29] for primary data

Conventional post-stroke rehabilitation efforts have had limited effectiveness in restoring the propulsion function inherent to a healthy bipedal gait, with functional improvements often being the product of compensatory mechanisms [26, 28, 30, 31]. The propulsion deficits that persist across the months and years post-stroke constrain long-term outcomes and contribute to a sedentary lifestyle, physical inactivity, and poor health [15, 32–37]. Indeed, post-stroke propulsion deficits are associated with a slow walking speed [17] and reduced long distance walking ability [38]—key predictors of real-world ambulatory activity in the home and community [33, 39, 40]. Examination of data reported in previous studies [17, 28, 29] demonstrates that functional speeds and distances are rare in those with little propulsion output from their paretic limbs. Indeed, people post-stroke who walk at the speeds and distances indicative of unlimited community ambulation (i.e., more than 7500 steps/d) [39] have relatively high levels of paretic propulsion (Fig. 1). More specifically, those who walk faster than 0.93 m/s—a walking speed that identifies individuals who walk more than 7500 steps/d with a specificity of 80% [39]—walk with an average peak paretic propulsion of 14.31 ±3.70%bodyweight (%bw) (Fig. 1a). Similarly, individuals able to walk farther than 288 m during the 6-minute walk test—a distance with similar discriminative abilities as a short-distance walking speed of 0.93 m/s [39]—walked with an average peak paretic propulsion of 10.90 ±3.62%bw (Fig. 1b). In contrast, those classified as home ambulators (i.e., individuals who walk less than 2500 steps/d) presented with substantially lower paretic propulsion of 3.55 ±2.38%bw and 3.33 ±2.51%bw, respectively.

In this expert review, we discuss recent advances in our understanding of post-stroke propulsion deficits, review emerging approaches to systematically diagnose and treat the underlying impairments, and highlight the substantial research and development effort that is required before these approaches can alter clinical practice. More specifically, the next section on “Propulsion diagnostics” overviews (i) the critical need for point-of-care propulsion diagnostics, (ii) the neurophysiological basis for propulsion impairments, (iii) the heterogeneous impairments underlying post-stroke propulsion deficits, and (iv) the potential for propulsion phenotyping to direct individualized therapies. The following section on “Propulsion treatments” overviews (i) the inadequacy of conventional intervention approaches and (ii) emerging propulsion-focused technologies and interventions that leverage internal and external mechanisms to target the different aspects of propulsion impairment.

## Propulsion diagnostics

In their 2017 consensus statement [41], the *Stroke Recovery and Rehabilitation Roundtable* recalls that the “most recent phase III and IV trials have been largely neutral or negative”, citing “considerable urgency” for “ensur[ing] that our trials are mechanistically well conceptualized, with careful matching of the nature of the intervention and the outcome measure chosen”. The international group went on to identify a core set of clinical outcomes to be assessed in every stroke recovery trial, but also acknowledged that clinical outcomes alone are unable to distinguish between restorative and compensatory recovery strategies. Their final general recommendation was that “trials need to consider serially applied kinematic/kinetic measurements alongside clinical assessments to distinguish between restitution and compensation”. The group highlighted the importance of measurements that reflect the “quality of hemiplegic gait performance” and the role that technology will play in making routine clinical assessment of kinematic and kinetic measurements feasible.

### Laboratory-based measurement of propulsion

The incorporation of force measurements in the analysis of human locomotion was first enabled in 1938 with the development of a mechanical force-reactive platform by Herbert Elftman [42]. Today, similar force measuring platforms can be found embedded within the instrumented treadmills and walkways widely used by motion analysis facilities and laboratories to study human walking. Modern force plate technologies leverage load cell sensors to measure the resultant orthogonal forces and moments that act on the force plate surface [43]. To measure propulsion (i.e., the anterior ground reaction force), the horizontally-directed forces are recorded using a multi-axis force plate. Multiple force plates are needed to measure the propulsion forces generated by each limb.

### Point-of-care propulsion diagnostics: if you can’t measure it, you can’t manage it

Despite the importance of propulsion to functional bipedal walking, the clinical management of impaired post-stroke propulsion is untenable without clinically-accessible approaches to characterize the extent and nature of paretic propulsion deficits. The lab-based tools traditionally used to assess propulsion (i.e., instrumented treadmills and force plates) are not accessible to most clinicians. These tools are also not viable for measuring propulsion function in real-world settings where people live and move. Although experienced clinicians may be able to grossly estimate a person’s propulsion function based on visual observation of gait kinematics [44], such expertise requires advanced training and years of practice. Moreover, modest changes in how the limb interacts with the ground (i.e., in terms of loading, duration, and orientation) may each be visually imperceptible, but together lead to changes in propulsion that have substantial effects on walking function. Indeed, the minimal detectable change for the peak of the anterior ground reaction force (i.e., peak propulsion) generated during walking is 2.85%bw [45]—i.e., only 5 pounds of force for a 175 pound individual. Even such modest changes in peak paretic propulsion are associated with meaningful changes in post-stroke walking function [29, 46–49].

Wearable sensors have rapidly gained popularity for rehabilitative applications [50]. These portable, low-cost, and unobtrusive measurement devices can provide objective, quantitative, and continuous information about motor behavior outside of the lab and in ecologically valid environments. Post-stroke applications of wearable sensor technology have ranged from assessment [51–54] to treatment [55]. A wide range of approaches for using wearable sensors to estimate ground reaction forces during walking have been proposed [56–59], with emerging focus on using minimal sensor sets to estimate key aspects of the propulsion forces generated by both healthy [60, 61] and post-stroke [61, 62] individuals. Further advances in hardware and computation that (i) provide key diagnostic information and/or (ii) reduce barriers to real world use would facilitate the translation of point-of-care wearable sensor solutions that can fill this crucial measurement gap. For example, multimodal hybrid sensors [50, 63] may allow monitoring both the kinematic (e.g., trailing limb angle) and neuromuscular (e.g., plantarflexor muscle activity) determinants of propulsion (see “Propulsion heterogeneity I: the interplay between kinematics and kinetics” section) and soft, textile-based sensors that can be integrated into clothing [50] may encourage better adherence when used for long-term monitoring in ecological settings.

### Neurophysiological basis for propulsion impairments

Symmetrical interlimb propulsion during walking requires the normal functioning of multiple circuits along the neuromotor axis. Stroke induces a cascade of neurophysiologic changes in cortical and spinal circuits that either directly or indirectly disrupt the corticospinal tract, the principal pathway for the control of voluntary, fractionated movements [64–69]. The overall strength of descending neural output to the motor system has been well-studied using transcranial magnetic stimulation (TMS) [70], and measures of corticospinal function and integrity have been linked to motor impairment and treatment effects [71–77]. In the context of post-stroke propulsion, individuals with more symmetrical corticomotor input to the plantarflexor muscles—the primary generators of positive power during walking—were reported to also show greater inter-limb symmetry in the generation of plantarflexor moments (see “Propulsion heterogeneity I: the interplay between kinematics and kinetics” section) during walking [78]. Further support for the importance of neural drive is found in a subsequent study demonstrating that a single gait training session targeting deficits in paretic propulsion through functional electrical stimulation of the plantarflexor muscles resulted in more symmetrical corticomotor input to the plantarflexor muscles after training that was related to more symmetrical plantarflexor moments across the paretic and nonparetic limbs [79].

Restorative therapies that aim to restore the normal functioning of the neural circuits affected by stroke may have strong potential to improve post-stroke propulsion. For example, neuromodulatory treatments such as repetitive TMS and transcranial direct current stimulation can be paired with gait training interventions to augment the excitability of the lesioned corticospinal pathways [80–84]. Paired associative stimulation has also been used to promote targeted plasticity in corticospinal circuits in individuals with stroke and spinal cord injury [85–87]. Ultimately, there is a critical need to rigorously test how approaches designed to target deficits in lower limb neuromotor control circuitry influence critical walking outcomes, such as paretic propulsion. However, the development of neurobiologically-informed, ‘top-down’ approaches that can be paired with activity-based rehabilitation interventions to target paretic propulsion deficits is hindered by several major gaps in our understanding of the neural circuit dysfunctions underlying specific post-stroke impairments. Indeed, beyond reduced output from the lesioned corticospinal pathway, stroke-induced abnormalities in non-corticospinal tract circuits—e.g., subcortical, brainstem, and spinal circuits—have been linked to post-stroke gait deficits and recovery, and merit further investigation.

Descending pathways emanating from non-lesioned cortical and subcortical circuits may show compensatory modulation of activity after stroke, and investigation of the role that neuromodulatory interventions can play in their function is warranted. For example, disrupted descending modulation of spinal circuitry may adversely affect post-stroke motor function. Indeed, increased excitability of spinal segmental reflexes, measured using H-reflexes, has been correlated with spasticity and excessive muscle coactivation after stroke [88, 89]. Similarly, hyperactivity in indirect descending pathways has been suggested to contribute to abnormal spinal excitability, spasticity, and movement synergies post-stroke [88, 90–93]. Moreover, indirect, brainstem-mediated corticofugal pathways (e.g., the reticulospinal and vestibulospinal tracts) modulate spinal excitability [88, 90–99] and appear to play an important role in the control of gait and posture. Recent evidence suggests that upregulation of these descending pathways may be an important mechanism underlying motor recovery after stroke [99, 100]. Indeed, that individuals with extensive corticospinal tract damage are able to maintain walking function [97] remarkably underscores the importance of indirect descending pathways to gait control. Similarly, corticospinal tract damage alone failed to predict changes in walking speed resulting from gait therapy, whereas incorporating other structures in the model predicted walking speed [71]. Other work showed that functional gains resulting from a treadmill exercise study were accompanied by increased activation of cerebellar and midbrain circuits [101], further highlighting the potential importance of subcortical structures to motor recovery. Further study of the role that neuromotor control pathways play in the generation of propulsion, and how gait therapies can be informed by neurobiology, is warranted.

### Propulsion heterogeneity I: the interplay between kinematics and kinetics

**Fig. 2:**
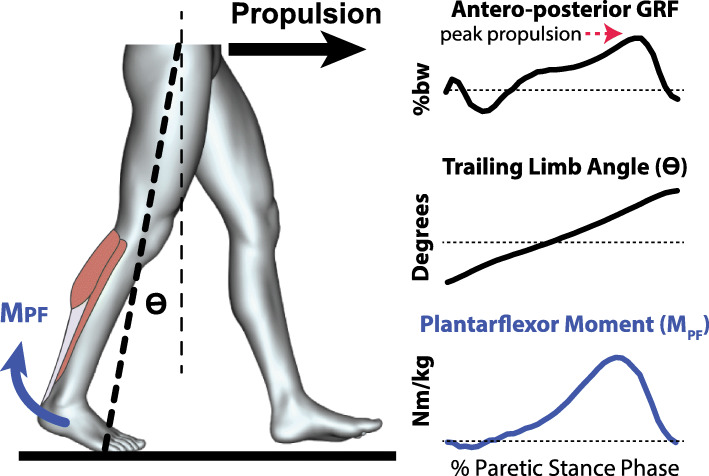
Forward propulsion results when a plantarflexor moment (M _*PF*_) is generated with the limb oriented behind the body [102, 103]

The individualization of propulsion-targeting gait therapies requires identification of the nature of the propulsion deficit and must thus account for the fact that both limb kinematics and kinetics affect propulsion [102, 103]. Indeed, propulsion is influenced by the trailing limb’s orientation to the body and the ankle moment generated by the plantarflexor muscles, with the trailing limb angle facilitating the translation of a plantarflexor moment into propulsion (Fig. 2 and see “Internal versus external plantarflexor assistance: limb angle matters!” section). Within this framework, propulsion deficits can be considered to be the result of primary deviations in limb positioning (i.e., deficits in joint range of motion, coordination, or balance), plantarflexor force generation (i.e., deficits in muscle coordination or strength), or a combination of the two. Indeed, people post-stroke may have range of motion restrictions (e.g., contractures of the lower extremity joints) that physically prevent the trailing limb posture required for a plantarflexor moment to translate to propulsion. Others may have full range of motion but cannot actively position the paretic trailing limb behind the body during walking (e.g., perhaps due to a balance deficit). Additionally, it is possible that adequate range of motion is available, but large ranges of hip extension are avoided to minimize disruptive heteronymous motor responses [104]. Still others may have the capacity and ability to achieve a normal trailing limb angle but are unable to activate the plantarflexors at the appropriate time or may simply not have sufficient plantarflexor strength. Impairments in any of these domains may result in patients adopting compensatory propulsive strategies. For propulsion-targeting therapies to be effective, they thus have to be well-matched to the specific needs of the individual. There is a need to study the complex interplay among biomechanical and neuromuscular determinants of propulsion, with the long-term goal of developing individualized therapeutic strategies to improve post-stroke locomotion.

### Propulsion heterogeneity II: central or muscular?

**Fig. 3:**
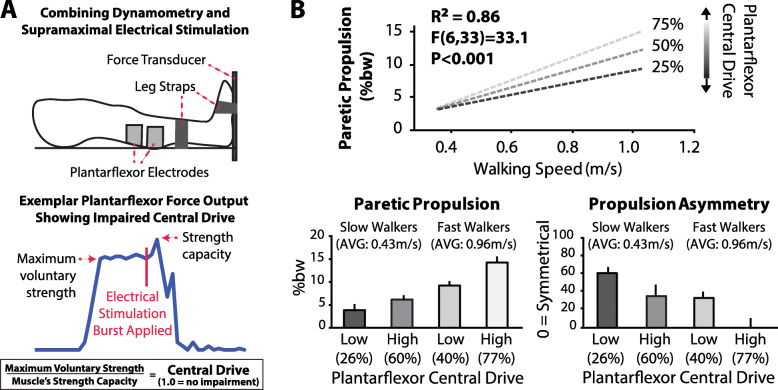
**a** Combining isometric strength testing with supramaximal muscle electrostimulation allows assessment of the extent and nature of post-stroke muscle weakness (i.e., maximum voluntary strength, strength capacity, and the ratio of these force measurements is the Central Drive). **b** Central drive is a key explanatory factor of paretic propulsion and propulsion asymmetry across individuals with a wide range of walking speeds. See primary source [105]

The heterogeneity of propulsion impairment is present at still deeper levels of analysis. The plantarflexor muscles are the primary generators of propulsive force during healthy walking, and post-stroke plantarflexor weakness may be the result of a reduced strength capacity (e.g., reduced physiological cross-sectional area due to muscle atrophy), reduced central neural drive, or a combination of these deficits. A promising diagnostic approach to elucidate the extent and nature of post-stroke muscle weakness combines dynamometry with supramaximal electrostimulation [105–107] (Fig. 3a). For example, the maximum voluntary plantarflexor force that community-dwelling individuals post-stroke are able to generate is only a fraction of their plantarflexor force-generating capacity, with the magnitude of this latent capacity shown to be a key explanatory factor of post-stroke propulsion impairments [105] (Fig. 3b). Deficits in voluntary plantarflexor force production have similarly been reported in older adults [108] and are thought to reflect changes in the central neural command to agonist muscles as opposed to muscle-level adaptations that may also be present [109].

Because individual patients may have a combination of reduced plantarflexor central drive and strength capacity that contributes to their propulsion impairment, assessing each of these potential deficits may be necessary to inform clinical decisions. However, the diagnostic systems currently used for neuromuscular function testing require substantial time to setup and execute, as well as costly and large equipment not widely available in clinical settings. Together, these factors motivate the development of novel point-of-care plantarflexor force measurement systems that can integrate neuromuscular electrical stimulation to assess the extent of and mechanisms underlying plantarflexor weakness.

A complementary assessment approach has recently emerged that leverages a posterior restraining force during walking to functionally assess an individual’s latent propulsion capacity, computed as the difference between an individual’s propulsion during unrestrained walking and their capacity to generate propulsion in the face of a restraining force—i.e., their “propulsion reserve” [110]. While potentially related, it is not clear if there is relationship between post-stroke central drive deficits to the paretic plantarflexors and the magnitude of a patient’s propulsion reserve, warranting further investigation. Ultimately, new diagnostic approaches that can systematically evaluate propulsion deficits and distinguish between a patient’s primary and secondary impairments [111] are necessary to advance individualized propulsion treatments.

### Propulsion phenotypes: towards individualized propulsion therapies

**Fig. 4:**
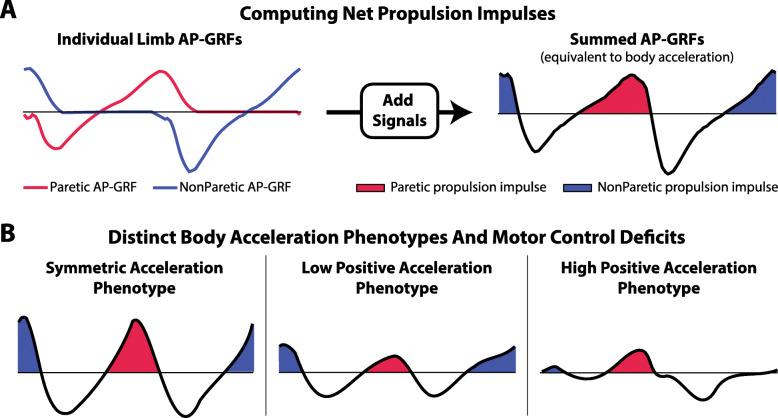
**a** The body’s forward acceleration during walking results from the interaction between the propelling trailing limb and braking leading limb. By summing the antero-posterior ground reaction forces (AP-GRF) generated by each limb, **b** distinct body acceleration profiles can be identified across individuals with different motor control deficits. The low acceleration subtype generates little forward acceleration during the paretic double support phase, whereas the high acceleration subtype demonstrates positive acceleration during paretic double support and then remains largely negative across the gait cycle

It has been suggested that features of post-stroke propulsion can be used to identify post-stroke gait phenotypes with different motor control deficits and that these phenotypes can be used to guide intervention matching [27, 112]. One approach computes the proportion of the total propulsion impulse generated by the paretic limb (Pp) such that a value of 0.50 indicates equal sharing of the propulsion load across limbs (Fig. 4). Using cutoffs equal to three standard deviations around the mean of healthy controls, we can classify individuals as having symmetric Pp, low Pp (i.e., ≤0.47), or high Pp (i.e., ≥0.53). These Pp classifications present with distinct body acceleration phenotypes during walking. Individuals with low Pp and high Pp both have reduced body acceleration during the double support phase of the paretic gait cycle compared to those with symmetric Pp (i.e., healthy controls); however, those with high Pp demonstrate positive acceleration during only paretic double support, whereas those with low Pp demonstrate relatively little positive acceleration during paretic double support [62] (Fig. 4b).

Crucially, individuals with low Pp present with motor control profiles that are different from individuals with high Pp. Indeed, the gait of individuals with high Pp is characterized by increased EMG activity of the extensor muscles during terminal stance, a shortened paretic step length, and prolonged paretic hip extension [113]. In contrast, the gait of individuals with low Pp is characterized by early and increased EMG activity of the flexor muscles, a lengthened paretic step length, and decreased paretic hip extension [114, 115]. With different underlying motor control profiles, individuals with low Pp and high Pp are likely to benefit from different targeted gait interventions; however, this hypothesis has yet to be validated and warrants further study.

Another approach to identify gait subtypes uses the peak paretic propulsion force (instead of the propulsion impulse) and combines this metric with a person’s walking speed to characterize individuals based on the combined knowledge of their walking performance (i.e., speed) and quality (i.e., peak propulsion). The co-assessment of these metrics was found to be substantially better at predicting the therapeutic response to a propulsion-targeting gait training program than either metric alone [112]. There may also be substantial value in the incorporation of neurophysiological and neuroimaging measures together with measurements of walking performance and quality to generate multi-modal propulsive phenotypes that can guide the selection of targeted treatment strategies individualized to a patient’s specific neuromechanical impairments. The concept of propulsion phenotyping is exciting but has limited translational potential without point-of-care measurement technology. Further investigation and validation of propulsion features with high prognostic value is required and may necessitate the identification of proxy measures of propulsion function that may be easier to estimate in clinical settings (e.g., see [116]).

## Propulsion treatments

### Conventional interventions do not target propulsion

Task-specific rehabilitation that emphasizes the direct practice of walking has emerged as a preferred approach to impairment-based training [117, 118]; however, walking practice that is not specifically structured to facilitate the recovery of a more physiological gait is likely to produce improvements via gait compensations [30, 119]. The current rehabilitation environment emphasizes the rapid attainment of walking independence, not gait restoration. Although walking independence is an important goal for stroke survivors, if independence is achieved via compensatory strategies, this inherently prevents the recovery of propulsion function needed for the fast, efficient, and stable gait characteristic of healthy bipedal locomotion.

In the face of persisting neuromotor deficits, passive walking aides are commonly prescribed to enable safe and independent walking. These devices, however, do not address deficits in paretic propulsion. Rigid ankle-foot orthoses (AFOs), for example, reduce drop foot during the paretic limb’s swing phase by constraining the ankle to a neutral position. By preventing plantarflexion during swing, the AFO enables safe ground clearance and reduces the risk of a fall; however, the other major role of a rigid AFO is to provide stability during the stance phase. In this role, the AFO may also limit plantarflexion during push-off, thus unavoidably limiting propulsion [6, 120, 121]. Ultimately, in compensating for persistent plantarflexor deficits, the positive power generated by the paretic limb is redistributed to favor hip-centric locomotor strategies [10, 13, 122, 123], preventing the recovery of normal propulsion function. An alternative to rigid AFOs are neuroprostheses. These devices use electrically-evoked muscle contractions to provide active assistance during functional activities. Although commercially-available systems have not been designed to target the plantarflexors and assist with propulsion, they are able to target the dorsiflexors and reduce drop-foot during the paretic swing phase [124, 125]. Because drop-foot neuroprostheses do not constrain ankle plantarflexion during the paretic stance phase, for appropriate individuals with the potential to recover propulsion function, neuroprostheses may be preferable to AFOs and may promote the recovery of walking function by way of gait restoration versus compensation.

In the last decade, technological and clinical advances have led to the development of novel rehabilitation programs and assistive devices that target post-stroke propulsion deficits. These have ranged from neuromodulatory interventions that facilitate activation of the impaired plantarflexors [79, 126, 127] to visual biofeedback interventions that guide individuals to propulsion-enhancing walking strategies [48, 128] to manipulations of different training parameters (e.g., load [129] or walking inclination [130]) that modify the propulsive demands of walking. More recently, wearable assistive robots that function in parallel with the underlying paretic musculature have been developed to *functionally* restore paretic propulsion deficits [47, 131, 132].

### Activity-based locomotor therapies

In the past 20 years, gait therapies that aimed to improve post-stroke walking function through repetitive stepping practice on a treadmill with body weight support and manual assistance, as needed, emerged as viable locomotor rehabilitation techniques [133]. However, in spite of numerous investigations, little is understood regarding *how* walking speed and functional performance gains were achieved by this intervention approach as the large trials did not collect kinetic and kinematic data. In a smaller sample study (*n*=15) of this rehabilitation approach that assessed all individuals with stroke as a single cohort, improvements in walking speed were observed without concomitant increases in propulsion symmetry [26]. Bowden *et al* additionally compared individuals who achieved clinically important improvements in walking speed (i.e., greater than 0.16 m/s) and individuals who achieved minimal gains (i.e., non-responders) to determine which factors were associated with changes in walking speed [28]. In this single arm, 27-person study of 12-weeks of locomotor training, the entire sample improved their usual, self-selected walking speed by, on average, 0.21 m/s; however, this increase was driven by treatment responders who had an average increase in walking speed of 0.27 m/s [28]. A substantial increase in propulsion symmetry was observed in the responder group (p=0.011) and was moderately correlated with changes in walking speed in the group as a whole (r=-0.47, p=0.014) [28]. Strikingly, propulsion was observed to trend towards becoming more asymmetric in the non-responder group, suggesting that an intervention’s ability to induce clinically-meaningful change may relate to improvements in paretic propulsion. This early investigation into how measures of propulsion differentiate individuals who respond and do not respond to activity-based locomotor therapies influenced future treatment approaches that innovatively sought to combine massed stepping practice with treatment elements targeting propulsion.

### The FastFES intervention

**Fig. 5:**
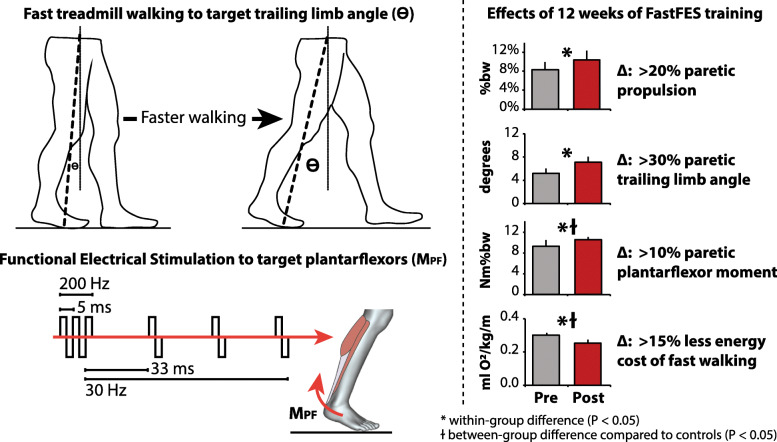
*Left-* The FastFES intervention targets deficits in paretic propulsion by combining fast treadmill walking (to increase the trailing limb angle) and FES to the paretic plantarflexor muscles (to increase the plantarflexor moment). *Right-* When compared to control training without FES at both fast and comfortable speeds, only FastFES training was shown to improve the paretic plantarflexor moment [31]. Interestingly, the two control groups also improved paretic propulsion, but did so by improving trailing limb angle. Consequently, only the FastFES training group reduced the energy cost of walking at both comfortable and fast walking speeds [119]

Traditionally, functional electrical stimulation (FES) is applied to the dorsiflexors to reduce drop-foot and facilitate ground clearance by the paretic limb. A novel training approach that applies FES to the plantarflexor muscles in combination with fast treadmill walking has emerged to target paretic propulsion deficits during walking (Fig. 5, Left). The FastFES intervention has been extensively studied, beginning with early findings that the combination of plantarflexor FES with fast treadmill walking was a potent combination to increase paretic propulsion [134]. A single arm, 12-person safety and feasibility study was then completed [46,135], showing the early promise of the FastFES intervention and informing the development of a therapeutic program. A 3-arm, 50-person randomized clinical trial to examine FastFES’ efficacy then compared 12 weeks of FastFES training to two control groups: 12 weeks of training without FES at either (i) comfortable or (ii) fast training speeds. Ultimately, it was shown that all three training groups achieved improvements in paretic propulsion and clinical measures of walking function (i.e., walking speed and distance); however, crucially, the two control groups that trained without FES achieved these gains primarily through a reliance on the paretic trailing limb angle, whereas the FastFES training group presented with durable therapeutic gains in both the paretic trailing limb angle and the paretic plantarflexor moment generated during walking [31]. Indeed, more recent mechanistic studies have revealed that FES-enhanced walking practice induces corticomotor plasticity and changes in muscle coordination that are not observed when training without FES [79,127,136*–*138]. Consequently, the FastFES training group was the only group to reduce the energy cost of walking at both comfortable and fast walking speeds [119] (Fig. 5, Right).

Interestingly, despite only the FastFES group presenting with a substantial reduction in the energy cost of walking, between-group differences in 6-minute walk test distance were not observed (i.e., all groups improved comparably) [119]. Future work is required to determine how improved propulsion control and a reduced energy cost of walking can be leveraged to reduce post-stroke walking disability. Indeed, while the FastFES body of evidence supports the importance of more normal propulsion function to an energetically economical gait [6], walking improvements made and measured in a motion analysis laboratory may not translate to improved walking in unconstrained, real world settings [139]. Overground adaptations of the FastFES training approach have the potential to increase ecological validity but require the development of new plantarflexor FES control approaches suitable for overground walking and the identification of methods to facilitate the necessary paretic trailing limb angle during training without the assistance of a fast treadmill belt.

### Propulsion-augmenting exoskeletons and exosuits

In contrast to FES, rigid exoskeletons and soft robotic exosuits have been developed to generate assistive torques *in parallel* with the underlying paretic muscles. Exoskeletons are rigid, brace-like structures that include on- or off-board actuation triggered and controlled by various sensors related to muscle function, joint kinematics, limb kinetics, and/or gait speed. Due to their rigid structure, they are capable of providing passive stability in non-actuated planes of motion. Recent advancements have allowed the creation of small and lightweight systems that can fit unobtrusively under or over clothes [140]. Most rigid exoskeletons are designed to enhance hip flexion and/or extension moments or the plantarflexor moment [131,132], presumably to target impairments in limb positioning and positive ankle work, respectively. Whereas the ankle exoskeletons are effective at responsively modulating plantarflexor moments to adjust for plantarflexor muscle activity [131] and gait speed [132], the impact on propulsion has been less encouraging. It appears that despite the increase in net plantarflexor moments, study participants concomitantly altered their limb posture such that this increased moment is not realized as greater propulsion [131,132]. It should be noted that “training” was not provided in this work. That is, device users were not taught how to make best use of the applied assistance. It is likely that the device alone, absent any training, may not produce the positive effects that are anticipated and speaks to the importance of therapeutic interventions so that patients can learn how to make best use of the applied assistance.

**Fig. 6:**
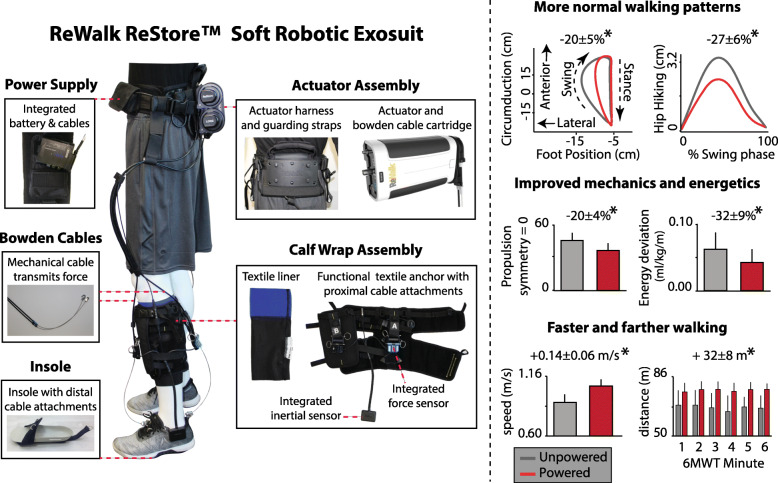
*Left-* A gait-restorative soft robotic exosuit commercially-adapted by ReWalk Robotics that recently gained FDA approval for use during stroke rehabilitation. *Right-* The exosuit technology was developed to assist both ankle dorsiflexion and plantarflexion function during post-stroke walking (see prior work [47, 143, 144, 147, 150])

In contrast to the work with rigid exoskeletons, exosuits are garment-like wearable robots consisting of functional textiles with integrated sensing and actuation. The first designs of soft wearable robots provided active support of impaired paretic plantarflexion and dorsiflexion function during hemiparetic walking [47]. The soft, lightweight, and unobtrusive human-machine interface of soft robotic exosuits uniquely allow for a natural interaction with the user in both powered and unpowered modes [47,141–143], enabling users to move about unrestricted when these devices are not active [143]. Preliminary studies conducted with small cohorts of community-dwelling individuals post-stroke showed an average 20 to 30% reduction in propulsion asymmetry [47], hip hiking and circumduction compensations [144], and the metabolic burden of hemiparetic gait [47,145]. Moreover, users self-selected faster speeds and walked farther distances when assisted by the exosuit [143]. As part of a successful application to the United States Food and Drug Administration, the ReStore^TM^ soft exosuit (ReWalk Robotics, Ltd., Marlborough, MA) (Fig. 6, Left) recently underwent a multi-site safety, device reliability, and clinical feasibility trial [146]. The trial recruited 44 users with post-stroke hemiparesis from across five clinical sites. Users participated in, on average, 311 minutes of treadmill and overground gait training with the device. Findings of no device-related falls or serious adverse events, high device reliability, and promising exploratory clinical findings complement the early laboratory research with device prototypes [47,144,145,147*–*149] (Fig. 6, Right) and motivate future controlled efficacy trials of this emerging wearable assistive technology.

### Internal versus external plantarflexor assistance: limb angle matters!

Despite the impaired state of the paretic plantarflexor muscles, they often retain a remarkable force-generating capacity [105]. This latent capacity can be exploited during propulsion-targeting gait training to facilitate the recovery of propulsion function. For example, robotic exoskeletons or exosuits can initially bypass this activation deficit to functionally restore plantarflexor forces during gait training, with the goal of tapering the assistance as a person’s underlying neuromotor function improves. In contrast, FES can be used to directly access this untapped muscle strength. It is likely that non-responders to one of these approaches may respond to the other, motivating further study. Alternatively, because exoskeletons/exosuits and FES are complementary force-generating approaches, there may be substantial value in exploring the integration of these technologies [151] for the treatment of post-stroke propulsion deficits. However, given that propulsion is dependent on both limb kinematics and kinetics (see Fig. 2), all active assistive devices that apply assistive plantarflexor forces must account for trailing limb angle deficits. Augmenting plantarflexor moments in a patient who cannot position their limb behind the body, or at the wrong time for an individual able to achieve an adequate trailing limb angle, would result in vertical, not forward movement of the body. Indeed, this consideration underpins the FastFES intervention’s combination of plantarflexor muscle FES with fast treadmill walking [46, 134]. Similarly, the onset timing of paretic plantarflexor assistance delivered by a soft robotic exosuit was shown to be a key factor in determining the subsequent effect on paretic propulsion, with plantarflexor assistance too early in the gait cycle even resulting in a reduction in paretic propulsion for some individuals [144]. Other investigators studying rigid exoskeletons have similarly posited the importance of considering trailing limb angle deficits when assisting plantarflexion [132].

### Body resistance for propulsion retraining

The approaches described above are intended to enhance gait by assisting patients to generate propulsion. The underlying assumption is that the individual does not have the capacity to increase propulsion on their own, requiring some means of external support (e.g., FES, robotics). In contrast, there is growing evidence that individuals with chronic hemiparesis can be made to access a latent propulsive reserve [152]. In particular, when individuals post-stroke are asked to walk faster [17, 153], step farther [154], or respond to visual feedback of their propulsion [48], they have the capability to increase propulsion. In fact, when faced with a posterior restraining force of up to 10 %BW during walking, the paretic limb was capable of increasing peak paretic propulsion by an average of 92%, with an increase in propulsive impulse of 225% [155]. These dramatic increases resulted in more symmetric propulsion and, importantly, persisted upon removal of the impeding force [155]. Importantly, the volitional increase in propulsion appears to arise from manipulation of limb posture (i.e., trailing limb angle) rather than an increase in the force output from the plantarflexors [155]—perhaps due to a reduced central drive [105] and/or impaired corticomotor input [78] to the paretic plantarflexors (see “Neurophysiological basis for propulsion impairments” and “Propulsion heterogeneity II: central or muscular?” sections). Nonetheless, the importance of limb posture during push-off arises from its representation as the anterior angle of the ground reaction force and is therefore highly correlated with peak propulsion [116].

The application of aiding and impeding forces to the body’s center of mass (COM) has a clear influence on propulsive forces and metabolic cost [129, 156]. Aiding forces that apply an anterior force to the COM can substitute for reduced propulsive limb forces but may contribute to neuromuscular slacking [157]. Because the imposed aiding force is substituting for the limb’s force, less muscle activity is required, and thus there is a decrease in the metabolic cost of walking [129, 156]. The timing of the aiding force on metabolic cost, however, is critical. Given the unilateral deficits in propulsion for individuals post-stroke, an aiding force is not required for both limbs. In fact, when a constant assistive force is provided to individuals post-stroke throughout the gait cycle, the metabolic cost of walking is not reduced. Instead, the metabolic cost of walking can be reduced when an imposed anteriorly-directed force applied to the COM coincides with paretic propulsion only. Reducing the metabolic cost of walking has important implications for locomotor duration and endurance, allowing for longer training. However, despite this improvement in the metabolic cost of walking, the paretic limb is producing less propulsion, suggesting that such a strategy will likely not lead to a patient producing greater propulsion after such training. Posterior impeding forces, however, will increase the cost of walking, but may create a suitable training environment because it encourages greater paretic limb propulsion.

### Propulsion biofeedback

Real-time biofeedback of propulsion function is a promising intervention approach to exploit the presence of a propulsion reserve in neurologically-intact [128, 158–160] and post-stroke individuals [48]. Biofeedback interventions enable individualized targeting of specific biomechanical impairments, provide focused practice of correct movement patterns by preferentially targeting the paretic leg, and capitalize on motor learning principles to optimize walking quality. Biofeedback can enhance an individual’s awareness of their gait impairment and enable self-correction of aberrant gait patterns [161]. Biofeedback has been used for modulating step length asymmetry [162] and muscle activity [163–166] in people post-stroke. More recently, in people with chronic post-stroke hemiparesis, treadmill training combined with visual and auditory feedback of propulsion function was shown to increase paretic propulsion and reduce propulsion asymmetry, with study participants improving both their trailing limb angle and plantarflexor moments during walking, as well as demonstrating short-term recall of the newly learned gait pattern [48, 158]. In addition, recent work demonstrates the efficacy of targeting propulsion deficits at the individual joint level, for example via real-time ankle power biofeedback [128]. Together, this early work demonstrates the feasibility and promise of propulsion biofeedback as a gait training strategy after stroke. Incorporation of wearable sensors to provide propulsion biofeedback or biofeedback about biomechanical variables that may be surrogates for propulsion (trailing limb angle, COM or shank acceleration [60, 62, 116]) during overground and community ambulation will further advance the effectiveness of gait biofeedback interventions. Additionally, consistent with the increasing popularity of ‘exergames’ and incorporation of gaming interfaces during rehabilitation, gamification of propulsion biofeedback can increase patient motivation, distract study participants from fatigue or boredom, and encourage greater repetitions during gait training [167–171].

## Conclusions

The extensive prior work showing a strong relationship between post-stroke propulsion and walking ability [27, 28, 38, 115, 129], coupled with the recent study of novel propulsion-targeting interventions and technologies [28, 47, 48, 119], highlight paretic propulsion as a key modifiable determinant of post-stroke walking function. In this expert review, we present the biomechanical and functional consequences of post-stroke propulsion deficits, review advances in our understanding of the nature of post-stroke propulsion impairment, and discuss emerging diagnostic and treatment approaches. In summary, post-stroke propulsion deficits are heterogeneous, existing diagnostic and treatment paradigms are not adequate, and emerging clinical and technological advances have shown substantial promise to help reshape the management of post-stroke propulsion deficits. Multidisciplinary teams of clinicians, engineers, and researchers are needed to translate existing lab-based diagnostic and treatment approaches to the clinic, as well as to develop the next generation of therapies and devices that will be enabled by technological progress in the areas of wearable technology and computational approaches. Clinical and technological advances in the areas of propulsion diagnostics and treatment will enable future rigorous testing of key neurorehabilitation hypotheses related to propulsion-restorative versus compensatory recovery paradigms, and ultimately the development of clinical practice guidelines capable of recommending diagnostic and treatment approaches based on the best available evidence.

## Data Availability

N/A.
